# Editorial: Natural products modulate the sensitivity of cancer to anti-PD-1 based immunotherapy

**DOI:** 10.3389/fphar.2023.1232456

**Published:** 2023-06-19

**Authors:** Jean Christopher Chamcheu, Qian Ba, Hang Ma

**Affiliations:** ^1^ School of Basic Pharmaceutical and Toxicological Sciences, College of Pharmacy, University of Louisiana, Monroe, LA, United States; ^2^ School of Medicine, Shanghai Jiao Tong University, Shanghai, China; ^3^ Bioactive Botanical Research Laboratory, Department of Biomedical and Pharmaceutical Sciences, College of Pharmacy, University of Rhode Island, Kingston, RI, United States

**Keywords:** natural products, immunotherapy, immune checkpoint blockade, cancer immunotherapy, anti-PD-1/PD-L1, sensitivity, resistance

Immunotherapy has emerged as one of the most promising therapeutic strategies for cancer treatments (Mellman et al., 2011). Among the immunotherapy approaches, immune checkpoint blockade (ICB) is regarded as a front-line immunotherapy for several types of cancer. Blockade of programmed cell death protein 1 (PD-1) and its ligand programmed cell death-ligands 1 (PD-L1) is one of the major ICBs that has been widely used to achieve cancer immunotherapy. Blockade of the interaction between PD-1 and PD-L1 can restore the immunological functions of T cells and exert their anti-tumor effects (Ohaegbulam et al., 2015). Although biologics including monoclonal antibodies (mAbs) targeting PD-1 or PD-L1 have been approved by the U.S. Food and Drug Administration (FDA) for different types of cancers, their application can be limited by drawbacks such as undesired off-target effects and prohibitive costs (Lin et al., 2020). On the contrary, small molecule-based PD-1/PD-L1 inhibitors may show advantages over mAbs, such as fewer side effects and more affordable cost (Wu et al., 2021). Small molecules, especially natural products and their derivatives, have been an important resource for the discovery of anti-cancer drugs. Indeed, a number of clinically approved anti-cancer agents are natural products-based small molecules with varied mechanisms of anti-cancer activity. However, only limited studies have been reported for the development of natural products-based anti-PD-1/PD-L1 agents. This was partially attributed to the challenges including a lack of suitable analytical methods for screening hits from natural products library and less well-characterized biological evaluations of natural products (Liu et al., 2021). Therefore, this Research Topic provides a snippet of published quality reviews of scientific literature and original research communication efforts on the discovery and development of natural products for ICB-based cancer immunotherapy. The published data presented will further increase our understanding of the role of natural bioactive ingredients and derivatives for sensitization of diverse cancers to ICB such as Anti-PD-1 Based Immunotherapy. While these published data highlight the natural products-derived molecules targeting diverse cancer molecular markers, they also provide promising mechanisms through which to reduce diverse human cancer burdens via targeting immune checkpoints and tumor microenvironments.

The first paper by Hernández-López et al., is a review that presents chimeric antigen receptor (CAR)-T cell therapy as a promising immunotherapy approach for cancer treatment and discusses the challenges faced by CAR-T cell therapy while highlighting the need for it to meet regulatory requirements (6). This review provides insights into the elements, history, and potential opportunities to improve CAR-T cell therapy, aiming to make it a widely accessible and effective treatment modality for cancer patients.

The second review by Dong et al. assessed the potential utility of natural products in overcoming the limitations of immunotherapy in colorectal cancer (CRC) treatment. While immune checkpoint inhibitors have shown benefits in certain CRC patients with dMMR/MSI-H, most CRC patients do not respond well to immunotherapy, partly due to internal resistance and immune escape. They discuss the advantages and highlighted the challenges in CRC treatment, explored the immunomodulatory effects of natural products and their bioactive components, and suggested that natural products hold potential as adjuncts in combined CRC immunotherapy approaches.

The third review by Wei et al., assessed, explored, and provided a comprehensive overview of the crucial role of gut microbiota in cancer development and the role of dietary fungi in cancer immunotherapy. They discussed the advantages of the manipulation of gut microbiota through direct implantation or antibiotic-based depletion and its impact on the overall effectiveness of cancer immunotherapies. They discussed extensively the biological functions, underlying mechanisms, and benefits of dietary fungal supplementation in promoting cancer immunotherapies through the modulation of gut microbiota.

The fourth paper by Kong et al., is a systematic review highlighting the current application and future potentials of astragalus polysaccharide (APS) in combination with cancer immunotherapy. APS is effective in activating adoptive immunotherapy, including lymphokine-activated killer (LAK) and dendritic cell-cytokine–induced killer treatment (DC-CIK) treatments, and regulates the PD-1/PD-L1 pathway and modulates cytokines, TLR4, NF-κB, MAPK pathways, and immune cells in the tumor microenvironment. They discussed that the combination of APS and immunotherapy holds great promise toward enhancing treatment efficacy as it activates immune responses while at the same time modulating the tumor microenvironment.

The paper by Chen et al. employed network pharmacology and molecular docking to investigate the components and mechanisms of *Solanum nigrum* L. (SNL) while providing insights into SNL’s therapeutic potential in the treatment of colon cancer. The study along with pathway analysis identified 37 SNL components, 796 target proteins, and 5,356 colon cancer genes, and determined that the key targets were mostly related to several signal transduction pathways, such PI3K-Akt signaling, drug response, and protein phosphorylation. By molecular docking, the study demonstrated the ability of SNL components, apigenin, and kaempferol, to bind the key target AURKB protein, while exerting anti-colon cancer properties.

In the study by Lam et al., YIV-906, a natural botanical cancer drug derived from traditional Chinese herbal formulation, demonstrates the ability to enhance T-cell activity and potentiate ICB and CAR T-cell therapies. YIV-906 was shown to activate T effector cells via upregulation of the expression of CD69, enhanced anti-PD1 therapy, and augmented CAR-T cell killing capability while inhibiting SHP1/2 activities as well as triggering downstream signaling pathways. These, therefore, suggest that YIV-906 is a potential immunomodulator in cancer treatment, supporting ICB and CAR-T cell therapy.

The study by Meng et al., investigated the potential of ginsenosides as inhibitors of TMPRSS2, a target for COVID-19 prevention and treatment. The role of TMPRSS2 was explored in cancer, focusing on lung adenocarcinoma (LUAD) patients, and its association with anti-PD-1 immunotherapy response and was highlighted that higher TMPRSS2 levels are associated with better prognosis in LUAD, but not in LUSC cohorts. Additionally, TMPRSS2 was found to be positively correlated with poor response to anti-PD-1 therapy, suggesting that TMPRSS2 could serve as a prognostic biomarker and a potential target for immunotherapy combination treatments in LUAD patients who are nonresponsive to anti-PD-1 therapy.

In a clinical trial study by Fukaya et al., they developed a population pharmacokinetics (popPK) and pharmacodynamics (popPK-PD) model for glucarpidase (CPG2) rescue treatment after high-dose methotrexate (MTX) therapy. This involved a phase 1 analysis in healthy volunteers and a phase 2 analysis in 15 patients who received CPG2 rescue for delayed MTX excretion. The population means pharmacokinetic parameters of MTX were estimated using the final model, and important sampling points for predicting plasma MTX concentrations at 48 h were identified. The study provided clinically significant insights into CPG2-MTX pharmacokinetics as well as Bayesian estimation of plasma MTX concentrations for effective treatment.

In a brief report by Li et al., they employed a functional assay (pair ELISA) and a PD-L1/PD-L1 binding assay (surface plasmon resonance; SPR) to evaluate a panel of natural products with previously reported anti-PD-1/PD-L1 activity and categorized based on their screening assays responses. The study provides insights into natural product-derived Immune Checkpoint PD-1/PD-L1 inhibitors binding capacities and blockade effects, thus emphasizing the role of using appropriate evaluation methods, including multiple-facet functional assays and target binding techniques.

Xie et al. investigated the role of myeloid-derived suppressor cells (MDSCs) in tumor progression and their modulation by granulocyte colony-stimulating factor (G-CSF) *in vitro* and in a neutropenic mouse model. They observed, *in vitro* an enhanced proliferation and immunosuppressive activity of MDSCs through the upregulation of gamma-glutamyltransferase (GGT) 1 by G-CSF. G-CSF administration *in vivo* with EL4 lymphoma resulted in increased MDSC numbers and attenuated the anti-cancer effect of chemotherapy. These suggested that aiming at GGT1 on MDSCs can thwart the tumor-promoting properties of G-CSF and proposed GGsTop as a potential combination agent during G-CSF treatment for febrile neutropenia in cancer patients.

Taniguchi et al. established murine models using human brain metastatic tumor cell lines and evaluate the effectiveness of perifosine, a bioavailable alkylphospholipid Akt inhibitor as a single agent in both ectopic and orthotopic models. Perifosine was found to be favorably distributed in the brain with prolonged localization, and significantly prolonged survival while inducing complete tumor regression in the orthotopic brain tumor mice group. Perifosine also exhibits strong antitumor responses against subcutaneous tumor growth and was associated with suppression of the PI3K/Akt signaling pathway, tumor cell proliferation, and inducing apoptosis. Therefore, perifosine represents promising for treating metastatic brain cancers.

Wang et al. investigated the occurrence of tumor lysis syndrome (TLS) associated with immune checkpoint inhibitor (ICI) therapies using real-world pharmacovigilance data. They reported that elderly male patients with lung and thymus malignancies were more susceptible to TLS. The onset time of TLS varied among different ICI therapies. The study emphasizes the need for caution regarding TLS as a potential adverse event of ICIs and highlights the importance of further monitoring in clinical practice.

Overall, the 12 papers published on this Research Topic highlighted several natural products that can be characterized appropriately to enhance immunotherapy of diverse human cancers. In summary, the intake of well-characterized natural products is a source of potential cost-effective therapeutics to treat cancer. They can be used in combination with other immunotherapies for the management of diverse cancer as chemopreventive, chemotherapeutics, or as adjuvant treatments. However, further studies using more physiologically relevant models and analytical tools are warranted to determine their efficacy and clinical potency. Therefore, further validation of these natural bioactives would improve cancer immunotherapy and reduce the burdens of advanced metastatic cancers.

## References

[B1] Lin X. Lu X. Luo G. Xiang H. (2020). Progress in PD-1/PD-L1 pathway inhibitors: From biomacromolecules to small molecules. Eur. J. Med. Chem. 186, 111876. 10.1016/j.ejmech.2019.111876 31761384

[B2] Liu C. Seeram N. P. Ma H. (2021). Small molecule inhibitors against PD-1/PD-L1 immune checkpoints and current methodologies for their development: A review. Cancer Cell. Int. 21 (1), 239. 10.1186/s12935-021-01946-4 33906641 PMC8077906

[B3] Mellman I. Coukos G. Dranoff G. (2011). Cancer immunotherapy comes of age. Nature 480 (7378), 480–489. 10.1038/nature10673 22193102 PMC3967235

[B4] Ohaegbulam K. C. Assal A. Lazar-Molnar E. Yao Y. Zang X. (2015). Human cancer immunotherapy with antibodies to the PD-1 and PD-L1 pathway. Trends Mol. Med. 21 (1), 24–33. 10.1016/j.molmed.2014.10.009 25440090 PMC4282825

[B5] Wu Q. Jiang L. Li S. C. He Q. J. Yang B. Cao J. (2021). Small molecule inhibitors targeting the PD-1/PD-L1 signaling pathway. Acta Pharmacol. Sin. 42 (1), 1–9. 10.1038/s41401-020-0366-x 32152439 PMC7921448

